# Sphygmomanometers and thermometers as potential fomites of *Staphylococcus haemolyticus*: biofilm formation in the presence of antibiotics

**DOI:** 10.1590/0074-02760160381

**Published:** 2017-02-16

**Authors:** Bruna Pinto Ribeiro Sued, Paula Marcele Afonso Pereira, Yuri Vieira Faria, Juliana Nunes Ramos, Vanessa Batista Binatti, Kátia Regina Netto dos Santos, Sérgio Henrique Seabra, Raphael Hirata, Verônica Viana Vieira, Ana Luíza Mattos-Guaraldi, José Augusto Adler Pereira

**Affiliations:** 1 Universidade do Estado do Rio de Janeiro, Faculdade de Ciências Médicas, Rio de Janeiro, RJ, Brasil; 2 Fundação Oswaldo Cruz-Fiocruz, Instituto Nacional de Controle de Qualidade em Saúde, Rio de Janeiro, RJ, Brasil; 3 Fundação Oswaldo Cruz-Fiocruz, Instituto Oswaldo Cruz, Rio de Janeiro, RJ, Brasil; 4 Centro Universitário Estadual da Zona Oeste, Laboratório de Tecnologia em Bioquímica e Microscopia, Rio de Janeiro, RJ, Brasil; 5 Universidade Federal do Rio de Janeiro, Instituto de Microbiologia Paulo de Góes, Rio de Janeiro, RJ, Brasil

**Keywords:** Staphylococcus haemolyticus, fomites, oxacillin, vancomycin, biofilm, pulsed-field gel electrophoresis

## Abstract

**BACKGROUND:**

The association between *Staphylococcus haemolyticus* and severe nosocomial infections is increasing. However, the extent to which fomites contribute to the dissemination of this pathogen through patients and hospital wards remains unknown.

**OBJECTIVES:**

In the present study, sphygmomanometers and thermometers were evaluated as potential fomites of oxacillin-resistant *S. haemolyticus* (ORSH). The influence of oxacillin and vancomycin on biofilm formation by ORSH strains isolated from fomites was also investigated.

**METHODS:**

The presence of ORSH on swabs taken from fomite surfaces in a Brazilian hospital was assessed using standard microbiological procedures. Antibiotic susceptibility profiles were determined by the disk diffusion method, and clonal distribution was assessed in pulsed-field gel electrophoresis (PFGE) assays. Minimum inhibitory concentrations (MICs) of oxacillin and vancomycin were evaluated via the broth microdilution method. Polymerase chain reaction (PCR) assays were performed to detect the *mecA* and *icaAD* genes. ORSH strains grown in media containing 1/4 MIC of vancomycin or oxacillin were investigated for slime production and biofilm formation on glass, polystyrene and polyurethane catheter surfaces.

**FINDINGS:**

ORSH strains comprising five distinct PFGE types were isolated from sphygmomanometers (n = 5) and a thermometer (n = 1) used in intensive care units and surgical wards. ORSH strains isolated from fomites showed susceptibility to only linezolid and vancomycin and were characterised as multi-drug resistant (MDR). Slime production, biofilm formation and the survival of sessile bacteria differed and were independent of the presence of the *icaAD* and *mecA* genes, PFGE type and subtype. Vancomycin and oxacillin did not inhibit biofilm formation by vancomycin-susceptible ORSH strains on abiotic surfaces, including on the catheter surface. Enhanced biofilm formation was observed in some situations. Moreover, a sub-lethal dose of vancomycin induced biofilm formation by an ORSH strain on polystyrene.

**MAIN CONCLUSIONS:**

Sphygmomanometers and thermometers are fomites for the transmission of ORSH. A sub-lethal dose of vancomycin may favor biofilm formation by ORSH on fomites and catheter surfaces.

The transmission of potentially pathogenic bacteria may occur via patient contact with a contaminated inanimate object or the hands of clinicians who have touched contaminated objects. Fomites include medical and non-medical devices, including sphygmomanometers, thermometers and stethoscopes (Cohen et al. 1997, Zargaran et al. 2015). Surface bio-contamination contributes to outbreaks of community-acquired and nosocomial infections through episodic fomite-mediated disease transmission and the persistence of fomite reservoirs. The extent to which fomites contribute to overall rates of nosocomial infection remains unknown. However, fomites play a notable role in the transmission of Gram-positive and Gram-negative bacterial pathogens, including staphylococcal species (Nascimento et al. 2015).

Fomite surfaces that are transported between hospital rooms are of particular concern and have been implicated in nosocomial outbreaks caused by different genera and species, including methicillin-resistant *Staphylococcus aureus* (MRSA) (Cohen et al. 1997, Nascimento et al. 2015, Zargaran et al. 2015). Since the 1970s, only a few studies have investigated the contamination of fomites by coagulase-negative staphylococci (CoNS), predominantly *Staphylococcus epidermidis* (Lowbury et al. 1971, Saito et al. 2014, Zargaran et al. 2015). Contaminated X-ray cassettes may serve as fomites for methicillin-resistant staphylococci (MRS) of varied pulsed-field gel electrophoresis (PFGE) types in hospital environments, including *Staphylococcus haemolyticus* (Kim et al. 2012). *S. haemolyticus* possesses multiple strategies for antimicrobial resistance, and thus options are limited among available antimicrobial agents (Kim et al. 2012). Patients with MRS infections are commonly treated with vancomycin (VA), which is potentially toxic and is administered only intravenously (Giormezis et al. 2014).

Among CoNS species, *S. haemolyticus* plays an important role in hospital-acquired opportunistic infections worldwide, including peritonitis, otitis, urinary tract infections, septicemia and prosthetic-device-associated infections (Giormezis et al. 2014). *S. haemolyticus* is also among the CoNS that colonise and cause bacteremia in neonatal intensive care units (ICUs) in many industrialised and developing countries, including Brazil (Mehta et al. 1991, Foka et al. 2006, Giormezis et al. 2014, Pereira et al. 2014). An analysis of the clonality of slime-producing methicillin-resistant CoNS (MR-CoNS) disseminated among neonates in Brazil (Pereira et al. 2014), India (Mehta et al. 1991) and Greece (Foka et al. 2006) revealed that most *S. haemolyticus* strains demonstrated multiresistance and produced slime.

Biofilm formation is recognised as an important form of growth that contributes to bacterial colonisation of and persistence on abiotic and biotic surfaces by many CoNS pathogens, including *S. haemolyticus* (Fredheim et al. 2009, Iorio et al. 2011, Giormezis et al. 2014, Pereira et al. 2014).

Therefore, it is essential to further investigate the environmental dissemination, virulence properties and molecular mechanisms of biofilm formation by *S. haemolyticus*. No studies conducted in Brazil have examined whether medical devices carry a risk of transmitting *S. haemolyticus* infection between subjects. Moreover, to the best of our knowledge, little information is available concerning the effects of antibiotics on the adherence/surface properties of *S. haemolyticus*.

*Objectives* - This study aimed to investigate the environmental survival of *S. haemolyticus* on abiotic sphygmomanometer, thermometer and stethoscope surfaces, which are common fomites in direct contact with clinicians, staff and patients (adults and neonates), in Pedro Ernesto University Hospital (HUPE teaching hospital), Rio de Janeiro, Brazil. Biofilm formation on catheter surfaces, antimicrobial susceptibility profiles and *mecA* and *icaAD* gene prevalence, which are associated with oxacillin (OXA) resistance and biofilm formation, respectively, were examined. In addition, the effects of minimal inhibitory concentrations (MICs) of VA and OXA on slime production and adherence properties on polystyrene and glass surfaces were investigated. Microorganisms were also analysed by performing PFGE assays to assess their clonal distribution.

## MATERIALS AND METHODS

*Origin and isolation procedures for CoNS strains* - The presence of *S. haemolyticus* on swabs taken from the surfaces of 37 medical devices (sphygmomanometers, thermometers and stethoscopes) from five hospital wards in a Brazilian urban hospital in August 2006 were assessed using standard microbiological techniques, as previously described (Cohen et al. 1997, Kim et al. 2012). Pedro Ernesto Hospital-University of the State of Rio de Janeiro (HUPE/UERJ) is a 600-bed tertiary teaching hospital with 5 ICUs, a renal transplant program and ambulatories that serve the metropolitan area of Rio de Janeiro, which has more than 6.32 million inhabitants.

Fomite samples demonstrated 100% bacterial contamination with CoNS. A total of 60 CoNS strains were isolated from the surfaces of 24 sphygmomanometers, 18 thermometers and 18 stethoscopes. Furthermore, 62.17% (n = 23) of the tested medical devices were colonised by two or three types of CoNS isolates. CoNS strains were stored in Trypticase Soy Broth (TSB; Difco Laboratories, Detroit, MI, USA) with 10% v/v glycerol at -20ºC in our laboratory.

Briefly, sphygmomanometers, thermometers and stethoscopes were previously wiped with a cotton swab moistened with sterile normal saline. The screening swabs were inoculated onto Mueller Hinton Agar (MHA) plates for 40 min, followed by post-collection and incubation at 35ºC for 48 h. Staphylococcus-like colonies were subcultured from each MHA plate onto 5% sheep’s blood agar media plates.

*Phenotypic identification and antibiotic susceptibility testing of S. haemolyticus strains* - Gram staining was performed on pure cultures of the isolates. All Gram-positive cocci were tested for catalase, DNase, coagulase activity, and growth on mannitol salt agar. CoNS were phenotypically characterised via a simplified method involving the following nine tests, as previously described (Iorio et al. 2007): two susceptibility tests using disks impregnated with 5 µg of novobiocin and 100 µg of desferrioxamine and tests to detect the production of clumping factor, pyrrolidonyl arylamidase (PYR), urease, and alkaline phosphatase, in addition to acid production from D-mannose, D-trehalose and D-xylose. The characteristics of the *S. haemolyticus* strains selected for analysis in this study are shown in Table II.

**TABLE II:** Microbiological and genetic properties of six *Staphylococcus haemolyticus* strains isolated from fomites, specifically sphygmomanometers (SF) and a thermometer (T), in the nosocomial environment of Pedro Ernesto University Hospital-University of the State of Rio de Janeiro (HUPE-UERJ), Rio de Janeiro, Brazil

Strains number and fomite	Hospital wards	PFGE types	*mec*A/*ica*AD genes	Antimicrobial multiresistance profiles*	MIC [µg/mL] (susceptibility)		Slime production on Congo Red Agar (CRA)		Adherence^#^ levels to abiotic surfaces			

									Glass binding assays		Polystyrene binding assays	
		
					OXA	VA	C	OXA/VA	C	OXA/VA	C	OXA/VA
11SF/9E3/06	Neonatal ICU	G_2_	- / +	I OXA,FOX,CIP,CLI,E,GEN,STX and CAZ, IPM, MXF	32 (R)	1 (S)	+	+ / +	+++	+++ / +++	++	++ / ++
SF/4BCGII/06	General surgery	I	- / +	I OXA,FOX,CIP,CLI,E,GEN,STX and CAZ, IPM, MXF	1 (R)	1 (S)	-	- / -	+++	+++ / +++	+++	+++ / +++
1SF/1BCG III/06	General surgery	J	- / +	II OXA,FOX,CIP,CLI,E,GEN,STX and CAZ, IPM	32 (R)	1 (S)	+	+ / +	+++	+++ / +++	-	+ / ++
28SF/2E2/06	General ICU	J	+ / -	III OXA,FOX,CIP,CLI,E,GEN,STX and CAZ	256 (R)	1 (S)	-	- / -	+++	+++ / +++	+	+ / +
33SF/4E5/06	Coronary ICU	G_1_	+ / -	III OXA,FOX,CIP,CLI,E,GEN,STX and CAZ	32 (R)	1 (S)	-	- / -	+	+++ / +	+	++ / +
27T/2BCG II/06	General ICU	H	- / +	IV OXA,FOX,CIP,CLI,E,GEN,STX	8 (R)	1 (S)	-	-/ -	+++	+++ / +++	++	++ / ++

C: assays performed without antibiotics oxacillin (OXA), vancomycin (VA); CAZ: ceftazidime; CIP: ciprofloxacin; CLI: clindamycin; E: erythromycin; FOX: cefoxitin; GEN: gentamicin; ICU: intensive care unit; IPM: imipenem; MIC: minimun inhibitory concentration; MXF: moxifloxacin; OXA: oxacillin; R: resistant; S: sensitive; SXT: sulfamethoxazole-trimethoprim; *: susceptibility to vancomycin (VA) and linezolid (LNZ) was observed for all strains tested; #: weakly (+), moderately (++), strongly (+++) adherent and (-) non-adherent to abiotic surfaces in absence (C) or presence of the antimicrobial agents - vancomycin (VA).

Antimicrobial susceptibility profiles were determined by the disk diffusion method according to the guidelines of the Clinical and Laboratory Standards Institute (CLSI 2012, Pereira et al. 2014), employing the following drugs: cefoxitin (FOX, 30 μg), gentamicin (GEN, 10 μg), sulfamethoxazole-trimethoprim (SXT, 25 μg), imipenem (IPM, 10 μg), ceftazidime (CAZ, 30 μg), ciprofloxacin (CIP, 5 μg), clindamycin (CLI, 2 μg), erythromycin (E, 15 μg), moxifloxacin (MXF, 5 μg) and linezolid (LNZ, 30 μg) (purchased from CECON, São Paulo, Brazil and Oxoid, Basingstoke, England). Any *S. haemolyticus* strains exhibiting a resistance phenotype to at least three different classes of antimicrobials were considered multi-drug resistant (MDR) strains (Giormezis et al. 2014).

MICs of OXA (Sigma, St. Louis, MO, USA) and VA (Oxoid, Basingstoke, England) were evaluated using the broth microdilution method (CLSI 2012). The concentrations ranged from 0.25 to 512 μg/mL for OXA and from 0.25 to 256 μg/mL for VA. The plates were incubated at 35ºC for 24 h (CLSI 2012, Pereira et al. 2014).

Bacterial stock cultures were maintained at -70ºC in 10% skim milk solution supplemented with 20% glycerol. The following microorganisms were used as controls for the phenotypic or antimicrobial susceptibility tests, biofilm formation or genotypic assays: *S. haemolyticus* ATCC 29970, *S. epidermidis* ATCC 35984, *S. epidermidis* ATCC 12228, *S. epidermidis* ATCC 14990, *Staphylococcus hominis* ATCC 27844, *Staphylococcus saprophyticus* ATCC 15305, *Staphylococcus warneri* ATCC 10209, *Staphylococcus aureus* ATCC 12600, *S. aureus* ATCC 29213, *S. aureus* ATCC 33591 and *S. aureus* ATCC 25923.

*Genotyping analysis* - *Multiplex PCR assay (mPCR) for the identification of methicillin-resistant S. haemolyticus* - mPCR to simultaneously identify *S. aureus*, *S. haemolyticus* and *S. epidermidis* species and to determine methicillin resistance based on the presence of the *mecA* gene was performed in accordance with previously described methods (Santos et al. 1999, Schuenck et al. 2008, Iorio et al. 2011). The primers and amplicons used in this study are listed in Table I.

**TABLE I:** Polymerase chain reaction (PCR) primers used in this study to identify *Staphylococcus haemolyticus* (SH), *S. epidermidis* (SE) and *S. aureus* (SA) species and to determine methicillin resistance based on the presence of the *mec*A (MRS) and *icaAD* genes, which play significant roles in slime production and biofilm formation

Primers	Sequence of forward and reverse primers 5’→ 3’	Product size (bp)	References
SH1	GGTCGCTTAGTCGGAACAAT	285	Schuenck et al. (2008)
SH2	CACGAGCAATCTCATCACCT		
SE1	CAGTTAATCGGTATGAGAGC	218	Iorio et al. (2011)
SE2	CTGTAGAGTGACAGTTTCGGT		
SA1	AATCCTTGTCGGTACACGATATTCTTCAGC	108	Pereira et al. (2010)
SA2	CGTAATGAGATTTCAGTAGATAATACAACA		
MRS1	TAGAAATGACTGAACGTCCG	154	Santos et al. (1999)
MRS2	TTGCGATCAATGTTACCTAG		
icaAD 1	GACAAGAACTACTGCTGCGT	546	Jong-Hyun et al. (2008)
icaAD 2	TACCGTCATACCCCTTCTCTG		

*PCR assay to determine the presence of the icaAD gene* - Experiments were performed in accordance with methods previously described (Jong-Hyun et al. 2008). The primers and amplicons are listed in Table I.

*Determination of clonal distribution by PFGE* - Genomic DNA was prepared using a method described previously (Pereira et al. 2014). DNA was cleaved with *Sma*I (New England BioLabs) according to the manufacturer’s instructions. PFGE was carried out in 0.5X TRIS-borate-EDTA-1.2% agarose gels at 13ºC in a CHEF DRII system (Bio-Rad). The pulse times ranged from 1 s to 35 s and were administered over the course of 23 h. Lambda DNA concatemers (New England BioLabs) were used as molecular size markers. Dice similarity coefficients were calculated with a band position tolerance of 1.5%, and the UPGMA method was applied for cluster analysis. Isolates were first assigned to PFGE types using 80% band-based similarity coefficients as cut-off values. Banding patterns were classified according to the criteria described by Van Belkum et al. (2007). Isolates exhibiting a similarity coefficient ≥ 80% were considered genetically PFGE-related. PFGE types were identified by letters, and subtypes were identified by letters followed by a numeric subscript.

*Slime production and abiotic surface adherence assays* - *Slime production on Congo Red Agar (CRA) medium* - Slime production was qualitatively detected by culturing the strains on CRA plates (CRA; Sigma Chemical Company, St Louis, MO, USA) as described previously (Chaieb et al. 2005, Pereira et al. 2014). Briefly, inoculated CRA plates were incubated under aerobic conditions for 24 h at 37ºC, followed by overnight incubation at room temperature. Slime-positive variants appeared as reddish-black colonies with a rough, dry, and crystalline consistency on CRA, whereas slime-negative strains appeared as pinkish-red smooth colonies with a darkening at the center. *S. epidermidis* strains ATCC 35984 and ATCC 12228 were used as positive and negative controls, respectively.

*Biofilm formation on a hydrophilic glass surface* - Microorganisms were inoculated in glass tubes (15x100 mm) containing 5 mL of TSB medium and incubated at 37ºC for 48 h. The supernatants containing non-adherent bacterial cells were discarded. Fresh sterile TSB (5 mL) was added to the test tubes and re-incubated for 48 h. This procedure was repeated twice. Glass-adherent bacteria created a confluent coat of cells on the sides of the tube. Microorganisms were classified as non-adherent (-: absence of adherence), weakly adherent (+: adherent bacteria appeared as a ring at the interface between the medium and the air), moderately adherent (++: bacteria attached on the side of the glass tubes), or strongly adherent (+++: bacteria attached on the side of the glass tubes and at the interface between the medium and the air). *S. epidermidis* strain ATCC 35984 was used as a positive control (Mattos-Guaraldi & Formiga 1991, Pereira et al. 2014).

*Biofilm formation on a hydrophobic polystyrene surface* - Semi-quantitative adherence assays were performed in sterile 96-well flat-bottomed plastic tissue culture plates (JET BIOFIL®) as previously described (Stepanovic et al. 2000, Pereira et al. 2014). Briefly, the strains were cultivated in TSB at 37ºC for 48 h, washed and re-suspended in fresh TSB with an optical density (OD) of 0.2 at 570 nm. Then, 200 μL of these suspensions were applied to microplate wells. After incubation at 37ºC for 24 h, the content of each well was aspirated and washed three times with 200 μL of phosphate buffered saline (PBS) at pH 7.2. The remaining attached bacteria were fixed with 200 μL of 99% methanol and stained with 2% crystal violet. The bound dye was then solubilised with 160 μL of 33% glacial acetic acid, and the solution OD was measured at 570 nm using an enzyme-linked immunosorbent assay (ELISA) plate reader (Bio-Rad, model 550). The cut-off OD (ODc) was defined as the mean OD of the negative control (TSB only). Based on the ODs of the bacterial films, all strains were classified into the following categories: non-adherent (0: OD ≤ OD_c_), weakly adherent (+: OD_c_ < OD ≤ 2x OD_c_), moderately adherent (++: 2x OD_c_ < OD ≤ 4x OD_c_), or strongly adherent (+++: 4x OD_c_ ≤ OD). Each assay was performed in triplicate and repeated three times. *S. epidermidis* strain ATCC 35984 was used as a positive control, and TSB medium was a negative control.

*Biofilm formation on catheter surfaces* - *Semi-quantitative biofilm formation on catheter surfaces* - Polyurethane 16-gauge percutaneous nephrostomy catheters (Intracath; Deseret Pharmaceutical Co., Sandy, Utah) were used to evaluate bacterial viability and biofilm formation on catheter surfaces. Sterile 4-cm segments of polyurethane catheters were immersed in TSB medium containing 10^6^ CFU/mL and incubated at 37ºC for 24 h (Gomes et al. 2009). The semi-quantitative roll plate technique (Maki et al. 1977) was performed on Columbia agar plates supplemented with 5% sheep’s blood (Oxoid, Germany) for 24 h at 37ºC.

*Scanning electron microscopy (SEM)* - Segments (1 cm) of polyurethane catheters infected in vitro with the *S. haemolyticus* 11SF/9E3/06 (PFGE type G_2_) strain were fixed with 2.5% glutaraldehyde and post-fixed with 1% OsO_4_, 5 mM CaCl_2_ and 0.8% K4[Fe(CN_6_)] in cacodylate buffer for 1 h at room temperature. Subsequently, the catheter segments were dehydrated in a graded series of ethanol, subjected to critical point drying with carbon dioxide, covered with a 10-nm layer of gold-palladium and examined with a JEOL JSM 5310 scanning electron microscope. Sterile unused polyurethane catheters were also processed by SEM immediately after removal from commercial packaging (negative control). Catheter segments infected in vitro with *Corynebacterium diphtheriae* CAT5003/BR were used as positive controls (Gomes et al. 2009).

*Influence of antibiotics on slime production and biofilm formation on abiotic surfaces* - *Effects of subMICs of OXA and VA* - The slime production and biofilm formation tests described above were performed using their respective media without antibiotics or with the addition of subMICs of OXA or VA equivalent to ¼ MIC, as previously described (Gomes et al. 2013).

## RESULTS

*Phenotypic and genotypic identification and epidemiological characteristics of S. haemolyticus strains* - As shown in Table II, six (1%) CoNS strains were identified as *S. haemolyticus* based on phenotypic tests and mPCR assays. Five *S. haemolyticus* strains were isolated from sphygmomanometers and one strain from a thermometer used in different ICUs and surgical wards of HUPE/UERJ.

**Fig. 1:**
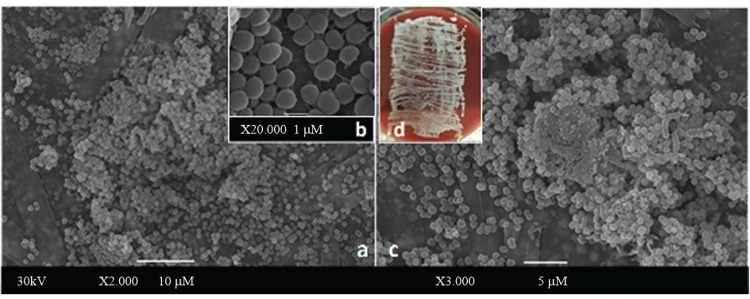
: biofilm formation on polyurethane catheter surfaces by oxacillin-resistant *Staphylococcus haemolyticus* (ORSH) isolated from fomites, verified by (A-C) scanning electron microscopy (SEM) assays and the roll plate Maki technique (D). After 24 h of incubation, the ORSH 11SF/9E3/06 [pulsed-field gel electrophoresis (PFGE) type G2; *icaAD*-positive] strain isolated from a sphygmomanometer used in the neonatal intensive care unit-Pedro Ernesto University Hospital-University of the State of Rio de Janeiro (ICU-HUPE-UERJ) (A) and grown in the absence of antibiotics produced a large amount of mature biofilm characterised by hollow voids on the luminal surface of the indwelling medical device; (B) detailed bacterial microcolonies indicative of a biofilm-producing pathogen; (C) biofilm production on catheter surfaces in the presence of ¼ minimal inhibitory concentration (MIC) of vancomycin (VA); (D) the roll plate assay indicated viable ORSH cells were extensively adherent (> 106 CFU) to and able to multiply on polyurethane catheter surfaces.

*S. haemolyticus clonal distribution* - The results of the PFGE assays are displayed in Fig. 1 and Table II. Five distinct PFGE types indicated genetic diversity among the *S. haemolyticus* isolates (n = 6) collected from the sphygmomanometers (n = 5) and thermometer (n = 1) used in different hospital wards. An identical PFGE type (J) was observed in two *S. haemolyticus* strains (1SF/1BCGIII/06 and 28SF/2E2/06) collected from sphygmomanometers used in a general surgery and a general ICU, respectively. *S. haemolyticus* (33SF/4E5/06 and 11SF/9E3/06) strains isolated from sphygmomanometers used in the coronary and neonatal ICUs, respectively, demonstrated a similarity coefficient ≥ 80% and were considered genetically related and consequently classified as belonging to PFGE subtypes G_1_ and G_2_.

*Antimicrobial susceptibility patterns and presence of the* mecA *gene* - As shown in Table II and Fig. 1, all six *S. haemolyticus* strains isolated from fomites were MDR to at least seven antimicrobial agents tested, regardless of their PFGE types and subtypes. All MDR *S. haemolyticus* strains were phenotypically resistant to OXA (ORSH) and showed susceptibility to only linezolid and VA. The OXA and VA MIC values were 1.0 µg/mL to 256 µg/mL and 1.0 µg/mL, respectively. The highest resistance level (profile I) was observed for *mecA*-positive ORSH strains (33.34%; n = 2) with OXA MICs ≥ 32 μg/mL belonging to PFGE types G_1_ and I. There was no evidence of the *mecA* gene in the majority (n = 4) of the ORSH isolates.

*Slime production and adherence to abiotic surfaces* - As shown in Table II, MDR ORSH strains (n = 6) isolated from fomites showed different levels of slime production and adherence properties to hydrophilic (glass) or hydrophobic (polystyrene) abiotic surfaces. The presence of the *icaAD* gene was detected in all *mecA*-negative ORSH (PFGE types G_2_, H, I and J) strains (n = 4). The *icaAD* gene was not detected in *mecA*-positive ORSH strains (n = 2).

Slime production on CRA medium was not observed for two *icaAD*-positive ORSH strains and two *icaAD*-negative strains. Different levels of slime production and adherence to glass or polystyrene surfaces were detected for ORSH strains. Biofilm formation on glass surfaces was detected for all (n = 6) ORSH strains, regardless of their PFGE type and slime production properties. The majority of these ORSH strains (n = 5) were classified as strongly (+++) adherent to glass surfaces. Biofilm formation on polystyrene surfaces was also observed for the majority (n = 5; 83.4%) ORSH strains but at lower levels than that observed for glass-binding assays. Positive results for all three tests were observed for only two ORSH strains. None of the strains were simultaneously negative for CRA, glass and polystyrene assays. The lowest adhesive properties were observed for the *icaAD*-negative ORSH strain (33SF/4E5/06 - PFGE type G_1_), which was slime-negative and weakly adherent (+) to both types of glass.

The presence of the *icaAD* gene was not correlated with slime production on CRA medium or adhesion to glass or polystyrene surfaces for MDR ORSH strains isolated from fomites.

**Fig. 2:**
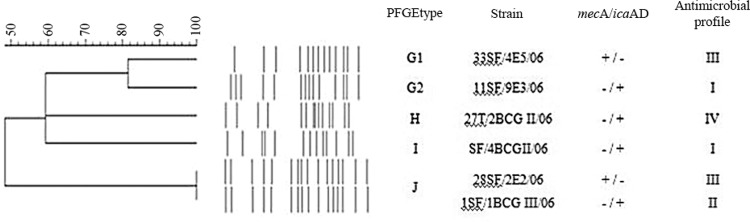
: pulsed-field gel electrophoresis (PFGE) profile dendrogram of the *SmaI*-digested genomic DNA of six oxacillin-resistant *Staphylococcus haemolyticus* (ORSH) strains isolated from two fomites, specifically sphygmomanometers (SF) and thermometers (T), in the nosocomial environment of Pedro Ernesto University Hospital-University of the State of Rio de Janeiro (HUPE-UERJ), Rio de Janeiro, Brazil. Similarity percentages are identified on the dendrogram derived from the unweighted pair group method using arithmetic averages and based on Dice coefficients. Microorganisms showing a similarity coefficient ≥ 80% were considered genetically PFGE-related. Multi-drug resistant ORSH (MDR ORSH) strains isolated from fomites utilised in different hospital wards demonstrated the following PFGE types: G1 [coronary intensive care unit (ICU)], G2 (neonatal ICU), H (general ICU), I (general surgery), and J (general surgery and general ICU). Polymerase chain reaction (PCR) reactions for the *mec*A and *ica*AD genes: +, positive; -, negative; antimicrobial multiresistance profiles: I to IV.

*Biofilm formation on polyurethane catheter surfaces* - Biofilm formation on catheter surfaces by ORSH strains isolated from fomites was verified by SEM assays and the roll plate technique (Maki test), as illustrated in Fig. 2.

*The influence of OXA and VA on slime production and biofilm formation on abiotic surfaces* - As shown in Table II, MDR ORSH strains isolated from fomites produced biofilms on abiotic surfaces in the presence of both OXA and VA. Biofilm formation levels of ORSH strains on glass and polystyrene surfaces in the presence of OXA and VA were not related to slime production or the presence of the *icaAD* and *mecA* genes. OXA and VA had no effects on slime production on CRA media by ORSH strains. Additionally, OXA and VA did not inhibit biofilm formation on glass and polystyrene surfaces by the ORSH strains of varied PFGE types evaluated in this study. In contrast, enhanced biofilm formation was observed in some situations. OXA enhanced biofilm formation on both glass and polystyrene surfaces by the ORSH 33SF/4E5/06 - PFGE type G_1_ strain. VA and OXA induced biofilm formation on polystyrene surfaces by the ORSH 1SF/1BCGIII/06 - PFGE type J strain. As shown in Fig. 2C, the production of mature biofilms with hollow voids on polyurethane catheter surfaces by ORSH grown in the presence of ¼ MIC VA was observed by SEM.

## DISCUSSION

Nosocomial pathogens may remain viable or persist on abiotic and biotic surfaces for months and thus are continuous sources of transmission in healthcare environments. Elimination of the sources and transmission of nosocomial pathogens remains a challenge, particularly in ICUs and surgical wards in developing countries. Incomplete cleaning procedures for equipment and patient rooms facilitate the transmission of MDR bacteria from one patient to another (Cohen et al. 1997, Kim et al. 2012, Zargaran et al. 2015). CoNS, particularly *S. epidermidis* and *S. haemolyticus*, cause bloodstream infections in approximately 37% of ICU patients, including those with indwelling medical devices (Giormezis et al. 2014). *S. haemolyticus* (90.3% ORSH; 70.4% MDR strains) of varied PFGE types was recently identified as the prevalent (77.5%) CoNS species associated with bacteremia in neonates making use of intravenous catheters in the neonatal ICU of HUPE/UERJ (Pereira et al. 2014).

In HUPE/UERJ, contaminated sphygmomanometers and thermometers, which are commonly in direct contact with patients or are transported between hospitals rooms, are fomites for ORSH of varied PFGE types that differed from the 6 PFGE types isolated from neonates. According to previous studies, MDR profiles may not be directly associated with resistance to OXA (Tabe et al. 2001, Pereira et al. 2014), but all ORSH strains isolated from fomites, independent of their PFGE types and subtypes, were characterised as MDR. The data indicated the adaptive ability of *S. haemolyticus*, including MDR ORSH strains, to circulate among different wards in this Brazilian tertiary-care hospital. In contrast to our findings, the majority of Korean ORSH strains isolated from X-ray cassettes were identical or closely related to each other according to PFGE analysis, suggesting a common source of contamination (Kim et al. 2012).

Similar to data previously described in the literature (Hussain et al. 2000), ORSH strains deprived of the *mecA* gene were observed in both neonates and fomites in the Brazilian nosocomial environment. The *mecA* gene was detected in 87.4% and 33.3% of *S. haemolyticus* strains isolated from neonates (Pereira et al. 2014) and fomites, respectively, independent of OXA susceptibility and PFGE type.

Biofilm formation was previously found to enhance the fomite survival of human pathogens. The medical importance of biofilms is attributable to their increased resistance to antimicrobial agents compared to that of free-living (planktonic) counterparts; consequently, diseases in which biofilms have a dominant role tend to be chronic and difficult to eradicate (Marks et al. 2014). Infections resulting from CoNS, including *S. haemolyticus,* most frequently occur after the implantation of medical devices and are associated with biofilm-forming potential (Lynch et al. 2007; Fredheim et al. 2009). Biofilm formation was also found to be a common phenotype among *S. haemolyticus* fomite isolates. Biofilm-forming ORSH strains are endemic in the Brazilian nosocomial environment (Pereira et al. 2014). Additionally, biofilm formation may enhance the fomite survival of ORSH. Because we demonstrated that a ORSH strain isolated from a fomite was able to produce a mature biofilm on the surface of a polyurethane catheter, we expect certain ORSH fomite isolates to be associated with the infection of implanted medical devices.

Biofilm development by CoNS is a multifactorial process (Fredheim et al. 2009). In *S. epidermidis*, bacterial adherence to a surface is mediated by a capsular antigen, specifically capsular polysaccharide/adhesin (PS/A), and bacteria multiply to form a multilayered biofilm associated with the production of polysaccharide intercellular adhesin (PIA), which is synthesized by *ica*-encoded proteins and mediates cell-to-cell adhesion and slime production. The presence of an *ica* operon in *S. haemolyticus* has been reported, but to date its contribution to biofilm formation remains unclear. Fredheim et al. (2009) demonstrated a clear difference in the biofilm structures of *S. haemolyticus* and *S. epidermidis*. In contrast to *S. epidermidis*, proteins and extracellular DNA are functionally relevant for biofilm accumulation, whereas PIA plays only a minor role. Therefore, the induction of biofilm formation and the determination of biofilm mass still must be optimised for *S. haemolyticus*. The ORSH *S. haemolyticus* strain isolated from fomites produced biofilm on both hydrophilic (glass) and hydrophobic (polystyrene) surfaces.

Although *ica*-positive strains showed high levels of biofilm production on glass surfaces, high levels of biofilm production on a glass surface independent of the *ica* gene were also observed in one situation. In addition, one *ica*-positive strain did not produce biofilm on polystyrene. Slime production on CRA medium was not observed for two *ica*-positive strains demonstrating high levels of biofilm production on glass and polystyrene surfaces. An *icaAD*-positive ORSH *S. haemolyticus* (11SF/9E3/06) strain isolated from a fomite that exhibited slime production on CRA medium and high levels of biofilm on glass and polystyrene surfaces was also able to produce mature biofilm with a thick and complex three-dimensional structure on the polyurethane catheter surface but only with small amounts of an extracellular matrix component. Although *icaA* and *icaD* play significant roles in biofilm formation, the presence of the *icaAD* gene does not always correlate with in vitro biofilm formation (Pereira et al. 2014). According to the present study, the biofilm formation process by ORSH fomite isolates is complex and may be unrelated to *ica* gene carriage. The biofilm-forming abilities of some isolates in the absence of the i*caAD* gene highlight the importance of further genetic investigations into *ica*-independent biofilm formation mechanisms. Moreover, some authors have suggested that the expression of biofilm phenotypes by ORSH may be regulated by several other factors, including environmental conditions (Fredheim et al. 2009, Pereira et al. 2014).

Little information is available regarding the effects of antibiotics on biofilm formation by *S. haemolyticus*. VA is an important anti-staphylococcal antibiotic. Although all ORSH strains isolated from fomites were highly sensitive to VA, biofilm formation, slime production, and adherence to hydrophobic plastic (polystyrene) and hydrophilic (glass) surfaces in the absence of antibiotics and in the presence the presence of ¼ MICs of VA or OXA were similar in most cases. Slime production on CRA by *icaAD*-positive and *icaAD*-negative strains was not altered by the presence of OXA or VA. Moreover, OXA and VA did not exert any inhibitory effects on biofilm formation on either glass or plastic surfaces by *icaAD*-positive and *icaAD*-negative ORSH strains. Interestingly, a sub-lethal dose of VA induced biofilm formation by an ORSH fomite strain (1SF/1BCG III/06 - PFGE type J) only on polystyrene, whereas OXA facilitated biofilm formation by two ORSH strains on both glass (n = 1) or polystyrene (n = 2) surfaces, regardless of the presence of the *icaAD* gene. As the majority of the strains (n = 4; 66.7%) demonstrated higher levels of adherence to hydrophilic glass than to hydrophobic polystyrene surfaces, both antibiotics and the type of abiotic material appear to influence biofilm formation. These findings with VA-susceptible ORSH strains were similar to those previously observed in studies examining the effects of sub-lethal doses of VA and OXA on biofilm formation by VA-resistant *S. aureus* (VRSA) (Zulfiqar et al. 2011). In contrast to VRSA, VA and OXA did not appear to exert any regulatory impact on *icaAD*, which is responsible for biofilm formation by OSRH strains isolated from fomites (Zulfiqar et al. 2011).

In conclusion, sphygmomanometers and thermometers are fomites for the transmission of various PFGE types of ORSH. A sub-lethal dose of VA favored biofilm formation by VA-susceptible ORSH on fomites. Therefore, VA susceptibility testing of planktonic bacterial cells based only on MIC values is ineffective for accurately determining the susceptibility of sessile bacterial cells. ORSH strains isolated from fomites were also able to produce biofilm on catheter surfaces in the presence of VA and are, therefore, potentially associated with invasive infections in patients with indwelling medical devices. Due to their ability to form biofilms on both hydrophilic and hydrophobic surfaces, ORSH strains of different PFGE types may potentially survive on various abiotic surfaces within a nosocomial environment. These data highlight the importance of *S. haemolyticus* as an emerging worldwide MDR nosocomial pathogen and the fact that different clones contaminating fomites may be responsible for nosocomial outbreaks, particularly in ICUs and surgical wards, including neonatal ICUs. Contamination barriers or decontamination procedures should be widely applied in developing countries. Further studies are necessary to define the roles of different components of *S. haemolyticus* biofilms and how they are regulated, particularly in the presence of therapeutic antimicrobial agents.
